# Ecological and human health risks associated with abandoned gold mine tailings contaminated soil

**DOI:** 10.1371/journal.pone.0172517

**Published:** 2017-02-21

**Authors:** Veronica Mpode Ngole-Jeme, Peter Fantke

**Affiliations:** 1 Department of Environmental Sciences, School of Ecological and Human Sustainability, College of Agriculture and Environmental Sciences, University of South Africa, Florida, Roodepoort, Gauteng Province, South Africa; 2 Quantitative Sustainability Assessment Division, Department of Management Engineering, Technical University of Denmark, Lyngby, Denmark; RMIT University, AUSTRALIA

## Abstract

Gold mining is a major source of metal and metalloid emissions into the environment. Studies were carried out in Krugersdorp, South Africa, to evaluate the ecological and human health risks associated with exposure to metals and metalloids in mine tailings contaminated soils. Concentrations of arsenic (As), cadmium (Cd), chromium (Cr), cobalt (Co), copper (Cu), lead (Pb), manganese (Mn), nickel (Ni), and zinc (Zn) in soil samples from the area varied with the highest contamination factors (expressed as ratio of metal or metalloid concentration in the tailings contaminated soil to that of the control site) observed for As (3.5x10^2^), Co (2.8x10^2^) and Ni (1.1x10^2^). Potential ecological risk index values for metals and metalloids determined from soil metal and metalloid concentrations and their respective risk factors were correspondingly highest for As (3.5x10^3^) and Co (1.4x10^3^), whereas Mn (0.6) presented the lowest ecological risk. Human health risk was assessed using Hazard Quotient (HQ), Chronic Hazard Index (CHI) and carcinogenic risk levels, where values of HQ > 1, CHI > 1 and carcinogenic risk values > 1×10^−4^ represent elevated risks. Values for HQ indicated high exposure-related risk for As (53.7), Cr (14.8), Ni (2.2), Zn (2.64) and Mn (1.67). Children were more at risk from heavy metal and metalloid exposure than adults. Cancer-related risks associated with metal and metalloid exposure among children were also higher than in adults with cancer risk values of 3×10^−2^ and 4×10^−2^ for As and Ni respectively among children, and 5×10^−3^ and 4×10^−3^ for As and Ni respectively among adults. There is significant potential ecological and human health risk associated with metal and metalloid exposure from contaminated soils around gold mine tailings dumps. This could be a potential contributing factor to a setback in the health of residents in informal settlements dominating this mining area as the immune systems of some of these residents are already compromised by high HIV prevalence.

## Introduction

Heavy metals and metalloids pollution of the environment remains a worldwide concern because of the negative effects that exposure to heavy metals can pose on various ecosystem and human receptors. Heavy metals and metalloids are introduced into the environment from geogenic (weathering) and anthropogenic sources including waste disposal, agricultural activities, vehicular traffic, petroleum refineries, paint industries, photography, and mining [1]. According to Kaasalainen and Yli-Halla [2], heavy metals emitted from anthropogenic origins including mining activities are highly mobile in the soil environment with increased potential to cause ecological and human health complications compared to those of geogenic origins. The contribution of metalliferous mining to elevated concentrations of heavy metals and metalloids including arsenic (As), cadmium (Cd), chromium (Cr), cobalt (Co), copper (Cu), iron (Fe), lead (Pb), mercury (Hg), manganese (Mn), nickel (Ni), uranium (U) and zinc (Zn) in the environment is widely reported [3–7]. Gold (Au) mining as a source of heavy metal contamination in soils has been documented in several countries including Korea [3], USA [4], Sultanate of Oman [6], Ghana [7], Canada [8] and South Africa [9]. Processing of ore bodies and disposal of mine tailings and wastewater rich in heavy metals and metalloids are the main origins of metal and metalloid release from Au mines [10, 11].

**Fig 1:**
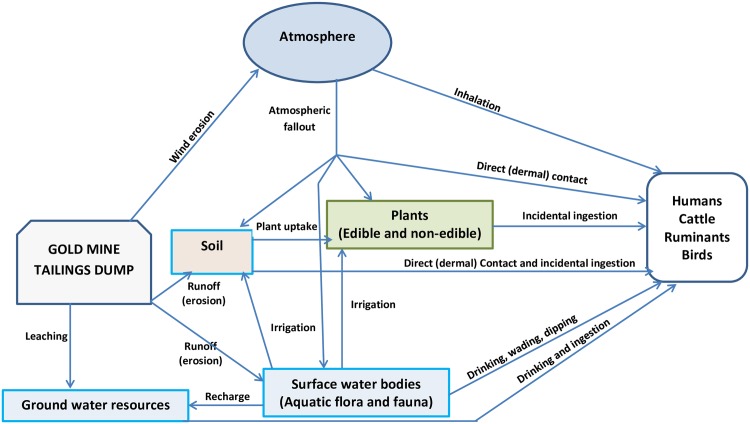
Conceptual illustration of the fate pathways of mining-related emissions of heavy metals and metalloids in the environment potentially leading to exposures for human and ecological receptors.

Metals and metalloids contained in mine tailings dumps are spread to various ecological receptors (fauna and flora), water resources, and the atmosphere when particles of the tailings are dispersed to surrounding environments through various environmental fate pathways (Fig 1). These metals and metalloids could be adsorbed by soil particles, taken up by plants, absorbed by micro-, meso- and macro-organisms, or leached to surrounding water bodies (Fig 1). In addition, tailings dumps are usually open, permitting the avian community and other animals to forage for prey which themselves may have been exposed to heavy metals and metalloids through dermal contact, and ingestion of contaminated food or particles or both [12]. The openness of the tailings also facilitates erosion which contributes to extensive spatial dispersion of the tailings particles and consequently heavy metals [13]. Tailings dumps therefore do not just affect the scenic view of the landscape but may also present significant risks to biotic and abiotic environments.

Many of the heavy metals and metalloids released from gold mining activities are toxic to plants and have the capacity to bioaccumulate, presenting health risks to humans, animals and ecosystems. The negative effects of metals on plants including oxidative stress, effects on fluorescence, stomatal resistance, chlorophyll and photosynthesis, reproductive processes, seed germination, seed morphology and seed physiology have been reported by Chibuike and Obiora [14]. Soil microbial activities are also negatively affected by high concentrations of heavy metals. Reduction in soil microbial population and distribution, and low microbial enzymatic activities in soil due to long-term exposure to heavy metals are some of the effects reported [15]. Arthropod populations as well as small and large mammals are also negatively affected by high levels of heavy metals in soils [16, 17]. Studies by Eisler [18] indicated that As doses of 17 mg/kg to 48 mg/kg body weight (BW) were fatal to birds, whereas some mammals were negatively affected by As doses of 2.5 mg/kg BW after oral exposure. Humans may be at risk via exposure to heavy metals and metalloids from Au mine tailings through dermal contact with the tailings and tailings contaminated soil, incidental inhalation and ingestion of contaminated suspended tailings and soil particles, ingestion of crops grown on mine tailings contaminated soils (Fig 1), or through deliberate ingestion of the tailings contaminated soils (geophagia). Poisoning as a result of heavy metal exposure is known to affect major human physiological systems including the skeletal, nervous, respiratory, excretory, and digestive systems. Whereas some of these heavy metals and metalloid (As, Ni, Cd, Cr, and Pb) are carcinogenic, they all have the potential to cause one or more health complications including skin damage, kidney disease, lung and nasal irritation and damage, fragile bones, nervous disorder cardiomyopathy, ulceration of stomach and small intestines, nausea, and decreased sperm count [19]. Heavy metal and metalloid pollution is likely to have significant implications for different ecosystems and the local human population living in the vicinity of mining activities.

Gold mining has been an important economic activity in South Africa for more than 120 years [20, 21]. The landscape of Krugersdorp, a city in the outskirts of Johannesburg in South Africa, is punctured by tailings dumps of abandoned and active Au mines. The present study was conducted in the vicinity of Tudor shaft, an abandoned shaft in Krugersdorp where an informal settlement has sprawled. This area has attracted both local and international environmental activists because of the high doses of radionuclides identified around the site. High levels of radionuclides with gamma dose rates of between 0.16 mSv/y and 0.25 mSv/y [22], and high concentrations of heavy metals and metalloids including As, Cd, Co, Cu, Hg, Mn, Ni, U, and Zn [23] have been reported around this area. Residents of informal settlements in the area grow vegetables for local consumption on the tailings dumps, which also serve as a playground for their children [22]. Health complaints that have been reported among residents in this community include dermatitis, flu, chronic cough, and wheezing chests [23]. Studies on assessment of human exposure to uranium in the area reported lifetime carcinogenic risk values as high as 1.01×10^−3^ [22]. Studies on abandoned and active mines in this area have so far focused on the levels of radioactivity and the concentrations of heavy metals in soils and water resources in the area. The ecological and health risk associated with exposure to the heavy metal and metalloid rich tailings and tailings contaminated soils have however not been investigated. Considering that HIV prevalence in South Africa is generally higher in informal settlements than in formal settlements and among the economically active age group [24], exposure of individuals with an already compromised immune system to heavy metals is likely to have direct consequences on the health of individuals in these communities as well as the economy and labor force of the country. The present study therefore aimed at determining the health risks associated with exposure of residents in the precincts of Au mine tailings dumps to heavy metals. Our study furthermore aimed at determining the potential ecological risks associated with the spread of heavy metals rich Au mine tailings to surrounding ecosystems.

## Materials and methods

### Description of the study area

**Fig 2:**
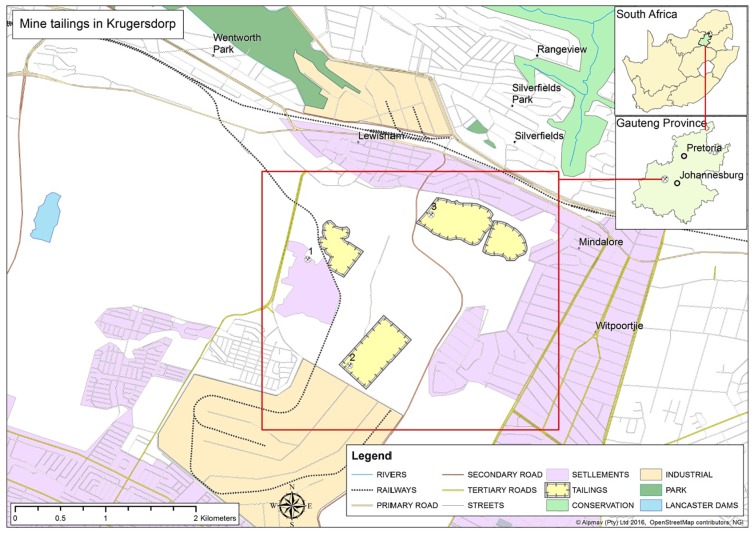
Study area in the Krugersdorp mining area with 1–3 indicating soil sampling sites.

The study was carried out in Krugersdorp (26°6’S and 27° 46’E), a mining city in the West Rand District of Gauteng Province (Fig 2), where Au, Mn, Fe, and asbestos have been mined over many years. The area covers a total of 247.2 km^2^ with a population of 140,643 and population density of 570 persons per km^2^. Geologically, the West Rand area of Gauteng is underlain by rocks of the Johannesburg Subgroup of the Witwatersrand Supergroup with outcrops of the Black Reef Formation of the Transvaal Supergroup [25]. The Witwatersrand Supergroup is made up of erosion resistant quartzites, ironstones and some marine lava deposits interspersed with softer, more easily eroded tillites, mudstones and conglomerates. Huge deposits of Au are found in some of the conglomerates of the younger members of the Supergroup [26]. Pyrite and uranite are among the minerals that have been associated with Au mining in the area. Decades of Au mining in the area has left more than 200 tailings dumps in the West Rand region where Krugersdorp is located. Information regarding the specific age of the individual tailings dumps in the area and the varying processes used to extract Au from the orebodies over the years is usually not available. The Tudor shaft community located on one of such dumps comprises of more than 2000 residents who are exposed to these tailings daily. The tailings in this area are bare of any vegetation and remain exposed to various agents of erosion permitting a wide spatial dispersion of tailings particles. Surrounding communities are therefore at potential risk from increased levels of heavy metal and metalloid exposure.

### Soil sample collection and characterization

Soil samples were collected within a 20 m radius of abandoned Au mine tailings dumps in three different areas in Krugersdorp located at 26° 7’ 56” S and 27° 48’ 13”E (Site 1), 26° 8' 34"S and 27° 48' 28”E (Site 2), and 26° 7' 40" S and 27° 48' 57"E (Site 3) (Fig 2). Gold mining in these sites had ceased more than a decade ago. A control sample was collected 6 km away from the study sites in an area with the same lithology but with little human interference. Soil sampling was performed in public spaces outside the borders of the mining activities. No formal permission was required to collect samples besides informing the occupants of the informal settlement near the mining areas. Neither endangered or protected ecological species nor humans were sampled for this study. All soil samples were collected using a plastic hand trowel at depths of 0 to 15 cm. Twelve samples were collected from both control and each study site once every month over a period of four months making a total of 48 samples per site. Samples were taken to the laboratory where they were characterized for their basic properties including particle size distribution and median diameter D(v,0.5), pH, organic matter content, electrical conductivity (EC) and cation exchange capacity (CEC). The particle size distribution of 24 samples from each site was determined using a Malvern Mastersizer 2000 laser particle size analyzer fitted with Hydro 2000G dispersion unit as described by Council for Geosciences [27]. The pH and EC of all samples collected were determined in a 1:2.5 (weight/volume) soil:water (distilled deionized) suspension, whereas organic matter content and CEC were determined using the modified Walkley Black and ammonium acetate techniques, respectively, as described by Van Reeuwijk [28]. The Student *t*-test was used to determine differences in means of the properties of soil samples from the different sites.

Whole rock mineralogy of 5 randomly selected samples from each site was determined using X-ray diffraction techniques. Quantitative analyses of minerals identified in the samples were preformed using the relative peak height/area proportions and reference-intensity-ratio (*RIR*) method where all diffraction data are scaled using a standard reference material (corundum) [29]. Identification of minerals from data and diffractograms obtained after scanning was carried out using the 2001 Version of the Philips X’PERT Graphics and Identify Software package. The mineral peak list produced by the software package was compared with those in the Mineral Powder Data File [30] for identification. Mean values of relative abundance of minerals in five samples from each site was presented as the relative abundance of the mineral in that site.

### Determination of heavy metals and metalloids in soil samples

Aqua regia (a mixture of 32% HCl and 55% HNO_3_ in a ratio of 3:1) was used to extract heavy metals in the samples as described by Lomonte et al. [31] and Goddard and Brown [32]. To 1 g of each sample weighed into a beaker, 10 ml of aqua regia was added and the mixture evaporated to near dryness in a water bath at 110°C. Another 15 ml of aqua regia was added to the mixture after which it was also evaporated to near dryness. 1M HNO_3_ was added to the mixture and the extract filtered through acid washed Whatmann no. 42 filter paper. The filtrate was collected and used for determination of heavy metals in each sample. A ContrAA 300 atomic absorption spectrometer was used in the determination of heavy metal concentrations in the sample extracts. For quality assurance and quality control purposes, two reagent blanks were included in each batch of samples analyzed. All samples were analyzed in duplicate. The reagent blanks were used as blanks in the determination of metal concentrations with the ContrAA 300. Values presented for heavy metals are therefore means of all samples analyzed per site. A Perkin Elmer Pure Plus multi element standard was used to determine percentage recovery of heavy metals analyzed. Percentage recovery of heavy metals and metalloids in the experiment ranged from 77% to 115% with average percent recovery being 86%– 107%. The lowest and highest percent recovery was obtained for As and Cu, respectively.

### Screening-level human toxicity and freshwater ecosystem toxicity assessment

As a first step, the potential of all involved heavy metals to pose a risk to humans and/or aquatic ecosystems was determined using the multimedia, multi-pathway model USEtox [33]. USEtox is the UNEP-SETAC global consensus model for characterizing chemical emissions in terms of human toxicity and freshwater aquatic ecotoxicity impacts [34]. It combines multimedia environmental fate, multi-pathway exposure and potential negative effects associated with a chemical emission into the environment in a set of characterization factors (CFs) that are expressed as:

- Increase in human cancer and/or non-cancer disease cases per kg emitted chemical into an environmental compartment (cases/kg) for human toxicity, and
- increase of the potentially affected fraction (PAF) of freshwater ecosystem species integrated over the exposed water volume and the duration of one day per kg emitted chemical (PAF m^3^ d/kg) for freshwater aquatic ecotoxicity,

In the present study, USEtox CFs for all relevant heavy metals for an assumed emission of 1kg/d into continental natural soil were used to contrast for As, Cd, Cr, Co, Cu. Mn, Ni, Pb and Zn and the contribution of different exposure pathways considered in USEtox to overall exposure for humans as well as to contrast the overall potential impacts on humans and/or the environment per kg emitted metal. USEtox however does not take into consideration dermal pathway of exposure in toxicity assessment

### Assessment of ecological risk from heavy metals and metalloids in tailings contaminated soils

Potential Ecological Risk Indices of As, Cd, Cr, Co, Cu. Mn, Ni, Pb, and Zn in the three contaminated sites were determined as directed by Håkanson [35]. Though the method was originally used to determine the ecological risk index for aquatic environments, studies by Qingjie et al [36], Zhu et al [37], Jiao et al [38], and Soliman et al. [39] have shown that the method is also applicable for assessing risks associated with heavy metal pollution in soils. The contamination factor of each heavy metal at each site (C_*f*_) and the degree of heavy metal contamination at each of the three sites (C_*deg*_) were determined according to Eqs (1) and (2), respectively. Potential ecological risk of each heavy metal was determined as indicated in Zhu et al [37] and Håkanson [35] using Eqs (3) and (4).

Cf=CoCn (1)

$$C_{deg}=\;\sum C_{f}$$ (2)

$$E_{r}=T_{r}\;\times C_{f}$$ (3)

$$RI=\;\sum E_{r}$$ (4)

Where C_o_ is the mean concentration of each heavy metal and metalloid in the soil sample, *C*_*n*_ is the background concentration of the element in the site (which in this case was taken as the concentration at the control site), *C*_*f*_ is the contamination factor for each heavy metal and metalloid, *C*_*deg*_ is the degree of contamination at each site based on studied metals and metalloids, *E*_*r*_ is the risk factor for each element, *T*_*r*_ represents the sedimentological toxic response for the given metal or metalloid, which according to Håkanson [35], Wu et al. [40], Soliman et al [39], and Jiao et al [38] are As = 10, Cd = 30, Co = 5, Cr = 2, Cu = 5, Mn = 1, Ni = 5, Pb = 5, and Zn = 1, and *RI* is the potential ecological risk.

### Assessment of human exposure to heavy metals and metalloid in tailings contaminated soils

The risk presented to both children and adults in the study area due to continuous exposure to mine tailings contaminated soil was determined as detailed in the Risk Assessment Guidance for Superfund Volume I Human Health Evaluation Manual (Part A), [41] Qu et al, [42], USEPA [41], and Kurt-Karakus [43]. Pathways related to three human exposure routes were considered in this study including (1) ingestion (2) inhalation, and (3) dermal exposure. Chemical daily intake (CDI) of each heavy metal and metalloid through each route was calculated as indicated in Eq (5) (ingestion route), Eq (6) (inhalation route), and Eq (7) (dermal route). Dermal exposure was taken as the sum over exposure pathways for different body parts, namely head, arms, hands, legs and feet. The values used in Eqs (5) to (7) are presented in Table 1. Results obtained from Eqs (5) to (7) were then compared with the documented reference dose (R*f*D) for each heavy metal analyzed as detailed in Agency for Toxic Substances and Disease Registry Toxic Substances Profiles [44, 45].

$$CDI_{\text{Ing}}=\frac{C_{UCL}\times IR\times CF\times FI\times EF\times ED}{BW\times AT}$$ (5)

$$CDI_{\text{Inh}}\;=\frac{C_{UCL}\times InhR\times EF\times ED}{PEF\times \;BW\times AT}$$ (6)

$$CDI_{\text{Dermal}}\;=\frac{C_{UCL}\times CF\times SA\times AF\times ABS\times EF\times ED}{BW\times AT}$$ (7)

**Table 1:** Health risk parameters and values used.

Parameters	Unit	Definition	Values used	
			Children	Adults
CDI	mg/kg/d	Chemical Daily Intake of heavy metal		
IR	mg/d	Ingestion rate	200	100
EF	d/year	Exposure frequency	350	350
ED	years	Exposure duration	6	30
PEF	----------	Particle emission factor	1.36x10^9^	1.36x10^9^
InhR	m^3^/d	Inhalation rate	7.6	20
BW	kg	Body weight	15	70
CF	kg/mg	Units correction factor	10^−6^	10^−6^
FI	----------	The fraction of soil or dust contacted that is presumed to be contaminated	1	1
AT	d	Average time over which exposure is averaged	EDx365	EDx365
SA	cm^2^	Skin surface area available for exposure	600 (head); 1060 (arms); 370 (hands); 1950 (legs); 490 (feet)	1250 (head); 2760 (arms); 960 (hands); 6400 (legs); 1300 (feet)
AF	mg/cm^2^	Adherence factor	0.04	0.02
ABS	----------	Dermal absorption factor	0.13	0.13

Sources: [41, 43, 45–48]

The values used for the concentration (C_*UCL*_) of the heavy metals and metalloids in Eqs (5)–(7) were the upper limits of the 95% confidence interval for the means of heavy metals and metalloids which according to USEPA [41], Hu et al. [49] and Zheng et al. [50] yields the maximum potential exposure for each heavy metal and metalloid considered. The averaged metal and metalloid concentrations of the samples from each site approximated a log-normal distribution; hence the data were transformed by using the natural logarithm of the heavy metal and metalloid concentration *ln*(*C*) [41]. The log-transformed data were then used to determine the UCL (upper confidence limit) for each metal and metalloid as described in USEPA [51], and Ferreira-Baptista and Miguel [47], as presented in Eq (8).

$$C_{UCL}=\;\text{exp}\left(\bar{x}+0.5s^{2}+\;\frac{sH}{\sqrt{n-1}}\right)$$ (8)

Where *UCL* is the upper confidence interval limit, *$\overset{-}{x}$* is the mean of log-transformed metal or metalloid concentration, *s* is the standard deviation of log-transformed data, *H* is the H-statistic, and *n* is the number of samples.

Non-carcinogenic risk posed by exposure of individuals in the community to the selected heavy metals and metalloids was determined by calculating the Hazard Quotients (*HQ*) as indicated in Eq (9) [42, 43]. According to Qu et al. [42], *HQ* values < 1 are assumed to be safe whereas *HQ* values > 1 present potential health risk associated with over exposure. The *HQ* values obtained were used to determine chronic hazard index (*CHI*) according to Qu et al. [42] and Ogunkunle et al. [52] (Eq (10). *CHI* values > 1 indicate a possibility of the occurrence of non-carcinogenic effects in individuals [43]. The carcinogenic risks associated with exposure to As, Cd, Co, Cr, Ni and Pb were determined as indicated in Eqs (11) and (12) [50, 53, 54]. A cancer risk below 1×10^−6^ is considered insignificant because 1×10^−6^ is classified as the carcinogenic target risk. A cancer risk above 1×10^−4^ is classified as unacceptable [41, 54]. Risk assessment parameters used in Eqs (9)–(11) are presented in Table 2.

$$\text{Non}-\text{Cancer Hazard quotient }\left(HQ\right)=\frac{CDI_{\text{route}}}{\text{chronic reference dose for element}\;\left(RfD\right)}$$ (9)

$$\text{Total chronic hazard index}\;\left(CHI\right)=\sum_{i=1}^{n}HQ=\;HQ_{\text{Ing}}+HQ_{\text{Inh}}+HQ_{\text{Dermal}}$$ (10)

$$\text{Carcinogenic risk}\;=CDI_{\text{route}}\times CSF_{\text{route}}$$ (11)

Where *CDI* represents the chemical daily intake for the exposure route determined as in Eqs 5, 6 and 7, and *CSF* represents the route-specific cancer slope factor indicated in Table 2.

$$\text{Total carcinogenic Risk}=Risk_{\text{Ing}}+Risk_{\text{Inh}}+Risk_{\text{Dermal}}$$ (12)

**Table 2:** Toxicological parameters for heavy metals and metalloids used in health risk assessment.

Heavy metal and metalloid	References doses (mg/kg/day)			Slope factors (mg/kg/day)		
	Inhalation	Ingestion	Dermal	Inhalation	Ingestion	Dermal
As	1.50E-05	3.00E-04	9.00E-04	15.1	1.50	3.66
Cd	1.50E-05	1.00E-03	1.00E-05	6.3	-	-
Co	6.00E-06	3.00E-04	1.60E-02	9.80	-	-
Cr	2.86E-05	3.00E-03	6.00E-05	42.0	-	-
Cu	6.90E-04	4.00E-02	1.20E-02	-	-	-
Mn	1.43E-05	4.60E-02	1.84E-03	-	-	-
Ni	9.00E-05	2.00E-02	9.00E-05	0.84	0.84	-
Pb	3.52E-03	3.50E-05	5.20E-04	-	8.50E-03	-
Zn	3.00E-01	3.00E-01	6.00E-02	-	-	-

Sources: [41, 43, 47, 49]

## Results and discussion

### Properties of soils around the tailings

**Fig 3:**
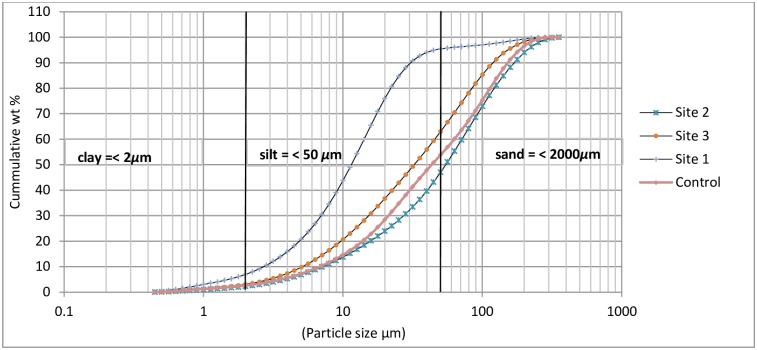
Mean particle size distribution of soil samples from each site (*n = 24*).

Mean particle size distribution of the soil samples differed from one site to the other as well as between the three study sites and the control site (P < 0.05). The mean particle sizes of soil samples from the three sites which varied between 0.4 μm to 355.7 μm (Fig 3) were smaller compared to particle sizes of soils from the control site (0.1 μm to 525.8 μm). With the maximum mean particle diameter of all samples being 355.7 μm, the soil particles at the study site could be described as being fine. Samples from Site 1 were finer than those from Sites 2 and 3 with clay sized-particles (< 2 μm) constituting 6.95%, 2.25%, and 2.95% of the volume of soil particles at Site 1, Site 2 and Site 3 respectively. Mean particle sizes of soils at the control site were 2.3% (clay), 48.6% (silt) and 49.1% (sand). Silt-sized particles constituted 88.5% of the soil particles at Site 1 and 44.8% and 60.1% of the soil particles at Sites 2 and 3 respectively. The median particle diameter of the samples according to values of D(v,0.5) were 44.8 μm, 11.3 μm, 56.4 μm and 31.7 μm for the control site, Site 1, Site 2 and Site 3 respectively.

Percival et al [8] also reported fine textured soils around Au mine tailings from Nova Scotia in Canada. The particle size distribution curves of the samples (Fig 3) indicate a narrow range of particles with values of coefficient of uniformity of 5.6 (Site 1), 9 (Site 2) and 8.4 (Site 3). With these values all being close to, or greater than 6, the soil particles can be described as being well graded [55]. Considering that host rocks are crushed to increase the surface area of the rocks in an endeavor to facilitate extraction of Au from the orebodies, the particles around the tailings are expected to be well graded.

Soils around the tailings had lower organic matter (OM) content (0.1% to 0.6%) than soils at the control site (0.3% to 1.88%). Low OM matter content in soils around tailings dumps is not unexpected because the environment is devoid of plant nutrients and the acidic nature of tailings would hinder vegetation growth, limiting the amount of organic matter in the soils. The soils around the tailings were more acidic (pH range from 2 to 6.23) with higher EC values (1.73 mS/cm to 11.52 mS/cm), compared to the soils from the control site with pH and EC ranges of 4.76 to 7.5 and 0.2 mS/cm to 1.15 mS/cm, respectively. The pH values observed in these soils are within the range of pH values observed by Nengovhela et al. [56] but much lower than those reported by Tariq et al. [57] in similar environments. Acidic conditions around Au mine tailings have been associated with high concentrations of pyrite contained in the tailings, which upon exposure, reacts with oxygen to form acid mine drainage. The acidic nature of the tailings would increase the mobility of potentially toxic heavy metals increasing their potential to cause harm to the environment. This would however depend on the CEC and mineral composition of the soils as these determine heavy metal mobility in the environment, and are both affected by the prevailing pH conditions. The CEC values for the soils were between 5.0 and 6.03 meq/100 g soil, whereas those at the control site were between 19 and 20 meq/100 g soil. Lack of organic matter, and low clay content would have contributed to the low CEC observed in the soil samples around the tailings. In addition, minerals identified in the soils (Table 3) have very low CEC.

**Table 3:** Mean relative abundance of minerals identified in samples.

Sample	Control Site	Site 1	Site 2	Site 3
Gypsum		< 1		
K-feldspar	-	-	-	1
Quartz	97	27	84	70
Mica	-	13	3	3
Kaolinite	3	5	-	1
Pyrophyllite	-	55	12	26

The mineral assemblage of soils from all three sites was dominated by quartz (Table 3). Samples from Site 3 had a more diverse mineral assemblage than those from Sites 1 and 2. Quartz constituted 70% to 84% of minerals in samples from Sites 2 and 3, whereas pyrophyllite made up 55% of the mineral constituents of samples from Site 1 (Table 3). The mineralogy of soils at the control site was dominated by quartz and kaolinite (Table 3). The dominance of quartzite in Witwatersrand Supergroup (bedrock of the study area) in addition to the resistance of quartz to weathering could explain the dominance of quartz in the samples. Gypsum (CaSO_4_. 2H_2_O) occurrence at Site 1 could be attributed to the presence of Ca in the tailings which might have resulted in its precipitation. No crystalline forms of Na, CN or Fe-bearing minerals were contained in the samples despite the commonly employed methods of processing which use Na-CN or K-CN as leaching agents. According to Percival et al. [8], Au mine tailings are usually fine textured with the mineralogy reflecting that of the host rock. Though pyrite constitutes part of the Witwatersrand Supergroup and has been reported in other Au tailings, it was not identified in this study possibly because of the shallow depth of sampling where pyrite might have been depleted as a result of oxidation after exposure to atmospheric oxygen. The mineral assemblage of the soils indicates a poor ability to sorb heavy metals, and an increased potential for ecosystem deterioration.

### Heavy metal and metalloid concentrations in soils around tailings

**Fig 4:**
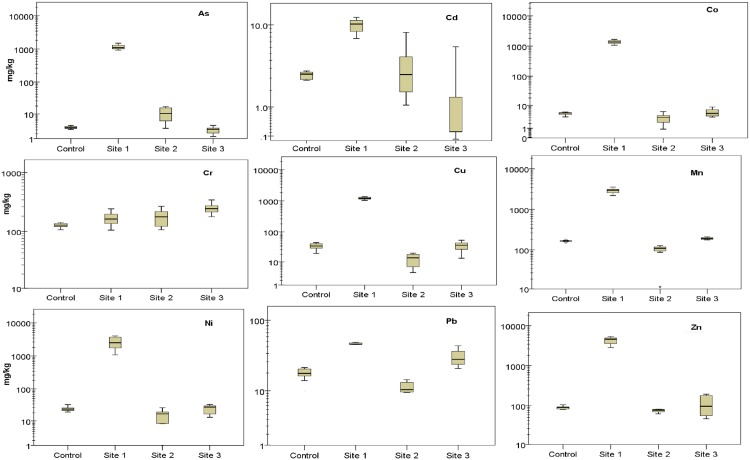
Concentrations of heavy metals and metalloids in samples of tailings contaminated soils.

The relative percent difference in heavy metal and metalloid concentrations between replicate analyses varied with sites. Detailed results of replicates are included in S1 Table. Though the study sites were all around Au mine tailings, the concentrations of metals and metalloids varied. Highest heavy metal concentration values were recorded in samples from Site 1, followed by those from Site 3, and then Site 2. Around Site 1, Zn had the highest concentration value (Fig 4) with a range of 2203 mg/kg– 5340 mg/kg whereas Cd had the lowest (1.1 mg/kg to 8.4 mg/kg). Values for heavy metal and metalloid concentrations in soil samples followed the order Zn > Mn > Ni > Co > Cu > As > Cr > Pb > Cd (Site 1), Cr > Mn > Zn > Ni > Cu > Pb > As > Cd > Co (Site 2), and Cr > Mn > Zn > Cu > Pb > Ni > Co > As > Cd (Site 3). These patterns were all different from the control site which had an order of Mn > Cr > Zn > Cu > Ni > Pb > Co > As > Cd (Fig 4). Except for Cr, heavy metal concentrations in samples from Site 1 were significantly higher than in samples from the control site (*P* < 0.05). Differences in heavy metal and metalloid concentrations in soils at Site 2, Site 3 and the control site were insignificant for As, Co, Cu, Mn, Ni and Zn (*P* > 0.05) but Cd, Cr, and Pb concentrations in samples from Site 3, were significantly different from those at the control site (*P* < 0.05).

**Fig 5:**
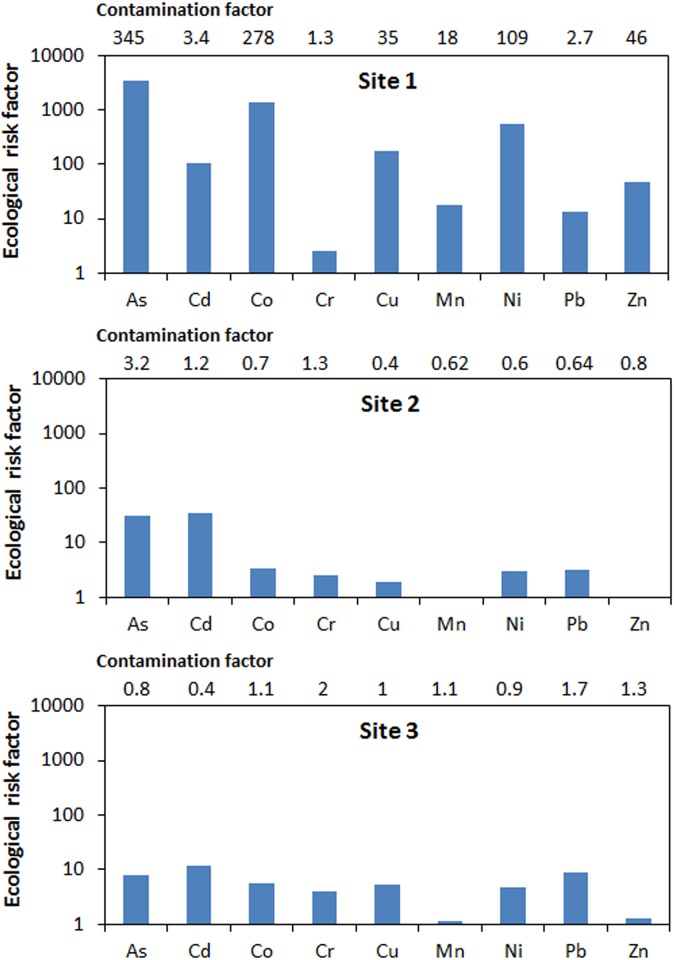
Heavy metal and metalloid contamination factors and ecological risk factors at the three tailings sites.

The values for some heavy metals like Cd and Cu in soils from the control site were on the high side of typical heavy metal concentrations reported in natural soils [58]. They however fall within the limits of screening values for heavy metals in soils in South Africa for all land use types protective of water resources, informal and standard residential areas [59]. Abdul-Wahab and Marikar [6], Meunier et al. [60], and Song et al. [61] reported similar heavy metal concentration values in Au mine tailings in their different studies. Despite these differences, results indicate significant enrichments of soils with heavy metals and metalloids as indicated by the values for heavy metal and metalloid contamination factors (Fig 5).

### Levels of heavy metals and metalloid contamination in soils around tailings dumps

Heavy metal and metalloid contamination factors *C*_*f*_ at the different sites ranged from 1.3 to 345 (Site 1), 0.4 to 3.2 (Site 2) and 0.4 to 2 (Site 3), (Fig 5, numbers on top of bars). At Site 2 and site 3, *C*_*f*_ values for Cr and As, respectively, were the highest whereas at Site 1, As had the highest C_*f*_ value. Contamination factors at Site 1 were highest among the three sites (Fig 5). According to contamination factor classification by Loska and Wiechula [62], levels of contamination observed for As, Cd, and Ni in soils at Site 3 were low (*C*_*f*_ ≤ 1), whereas contamination levels for Co, Cr, Cu, Mn, Zn and Pb were medium (1< *C*_*f*_ ≤ 3).

Heavy metal and metalloid contamination levels of soils at Site 2 were low for Co, Cu, Mn, Ni, Pb and Zn, medium for Cr and Cd, and high for As (Fig 5). Soils at Site 1 were more contaminated with contamination levels for Cr and Pb classified as medium and As, Cd, Co, Cu, Ni, Mn and Zn classified as high (Fig 5). The degree of soil contamination at the three sites therefore varied being very high for Site1 (8.4x10^2^), low for Site 2 (9.3), and medium for site 3 (10.3) based on the classification of *C*_*deg*_ values by Loska and Wiechula [62] where *C*_*deg*_ < 5 indicate low pollution; 5 ≤ *C*_*deg*_ < 10, medium pollution; 10 ≤ *C*_*deg*_ < 20, high pollution; and *C*_*deg*_ ≥ 20, very high pollution. Increased level of heavy metal and metalloid contamination around these sites might have occurred as a result of leaching from the tailings or dispersal of the tailings particles to surrounding environment. Dispersal by wind would have been facilitated by the silky texture of the particles. Surrounding ecosystems are therefore at risk of heavy metal exposure from contaminated soil.

### Screening-level human and freshwater ecosystem exposure and toxicity impacts

**Fig 6:**
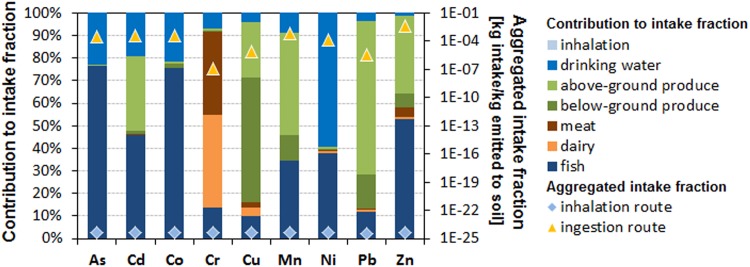
Distribution of pathways contributing to potential human exposure following an assumed emission of different heavy metals and metalloids of 1 kg per day to natural soil.

**Fig 7:**
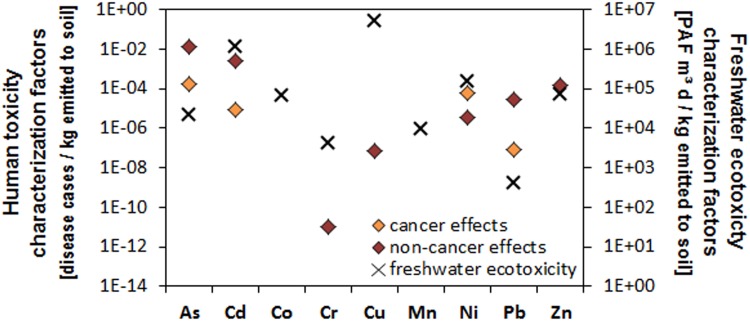
Distribution of potential human toxicity (left y-axis) and freshwater ecotoxicity (right y-axis) impacts following an assumed emission of different heavy metals and metalloids of 1 kg per day to natural soil.

Exposure to metals released to natural soil is many orders of magnitude higher via the ingestion route (including pathways for ingestion of drinking water, above-ground produced crops and below-ground produced crops like root and tuber crops, meat, dairy and fish) than via the inhalation route across heavy metals (Fig 6). Ingestion-related pathways contribute differently to overall ingestion exposure and are dominated by ingestion of fish mainly following runoff and leaching from soil to freshwater contributing up to 77% to overall human exposure for As and Co, followed by above-ground produced crops (up to 68% contribution for Pb) and below-ground produced crops (up to 55% contribution for Cu) mainly following root uptake into crops, and drinking water ingestion (up to 60% contribution for Ni). The exception is Cr, where dairy (41%) and meat (37%) consumption are the main contributors to human ingestion exposure assuming that emissions refer to trivalent chromium, which binds more strongly to organic matter than most other metals. Combining human ingestion and inhalation exposure with effect factors relating human exposure to aggregated potential cancer and non-cancer effects in the USEtox model, characterization factors for human toxicity were derived and are shown in Fig 7.

Highest toxicity potentials have As and Cd with 1x10^-2^ and 2x10^-3^ cumulative non-cancer cases in an exposed population per kg emitted to natural soil, respectively, while for cancer effects, highest toxicity potentials are seen for As, Ni and Cd with 2x10^-4^, 6x10^-5^ and 9x10^-6^ cases per kg emitted, respectively. Similarly to human toxicity, the mass fraction of metal emission to soil reaching freshwater was combined with freshwater aquatic ecotoxicity effect factors in USEtox to quantify characterization factors for freshwater ecotoxicity. Highest freshwater ecotoxicity potentials are seen for Cu, Cd and Ni with respectively 5.2x10^6^ and 1.2x10^6^ and 1.5x10^5^ PAF m^3^ d per kg emitted to natural soil.

Despite large variability of toxicity and ecotoxicity results between metals, there are also large uncertainties related to toxicity characterization, ranging from one to two orders of magnitude for freshwater ecotoxicity to more than two orders of magnitude for the more complex ingestion-related exposure pathways for human toxicity [33]. From these screening-level results, a more detailed assessment of potential risks is presented in the following sections starting from the actual heavy metal concentrations in soils around the mining sites. However, USEtox characterization factors are normalized to emitted mass and would have to be adapted for use with measured metal concentrations in soil. More specifically, soil concentrations would need to be combined with considered soil volume and bulk density to obtain metal mass in soil, which could then be set as either initial conditions (in case of dynamic characterization) or continuous emission when combined with the residence time in soil varying significantly between metals (in case of steady-state characterization). Since soil volume and residence time are not available in the current study, site-specific potential risk was estimated using contamination and risk factors as described in the following.

### Ecological risk associated with heavy metals and metalloids in mine tailings contaminated soils

In soils from Site 1, *E*_*r*_ values ranged from 2.6 for Cr to 3.5 x 10^3^ for As (Fig 5). E_*r*_ values for soils from Site 2 were lower with a range of 0.6 (Mn) to 35.4 (Cd). Manganese also had the lowest *E*_*r*_ value (1.1) in soils from Site 3 and Cd the highest *E*_*r*_ value (11.59). Although Cd concentrations were very low in the samples, the ecological risk for Cd was high because of the higher toxic response value (*T*_*r*_) of Cd compared to the other metals. There was also a high ecological risk of Cd at the control site because of the high concentrations of Cd observed at the site. This is also confirmed by results from screening with the USEtox model, which indicates that Cd presents a high potential ecotoxicity risk per emission unit. The values of some of the heavy metals like Cd, Cu and Pb in the soils from the control site were higher than those in soils around the tailings. The occurrence of high concentrations of heavy metals at the control site might have occurred as a result of dispersal of the tailings particles by wind, which is prevalent in the study area especially during the months of August and September. These metals could also have originated from other anthropogenic activities. Though the observed values for heavy metals are higher than the average for most soils [58], they fall within the range of screening values for soils in South Africa [59]. These results also indicate that there may be heavy metal associated ecological risk even at the control site. The Ecological risk of a metal can be classified as low when risk factor (*E*_*r*_) ≤ 40, moderate when 40 < *E*_*r*_ ≤ 80, considerate when 80 < *E*_*r*_ ≤ 160, high when 160 < *E*_*r*_ ≤ 320 and very high when *E*_*r*_ > 320 [37], [63], [64]. Values for ecological risk factors for the various heavy metals and metalloids in soils from the three study sites were increasing in the following order: from Cr (lowest), Pb, Mn, Zn, Cd, Cu, Ni, Co to As (highest) for Site 1; from Mn (lowest), Zn, Cu, Cr, Ni, Pb, Co, As to Cd (highest) for Site 2; and from Mn (lowest), Zn, Cr, Ni, Cu, Co, As, Pb to Cd (highest) for Site 3. Potential ecological risk was highest at Site 1 (*RI* = 5.7x10^3^) followed by Site 2 (*RI* = 82), and then Site 3 with an *RI* value of 50. This classification is according to ranges presented by Zhu et al. [37], Håkanson et al. [63], and Chen et al. [64] where *RI* ≤ 150 represents low potential ecological risk, 150 < *RI* ≤ 300; moderate ecological risk potential, 300< *RI* ≤600; considerable ecological risk, and *RI* > 600; very high ecological risk. Our results indicate high risks to biodiversity in the area from soil contamination with metals. High concentrations of these metals and metalloid in the soil would likely affect colonization of the area by vegetation which may reduce the organic matter content in the soils, and their ability to retain metals. This would also render the area more prone to erosion. There will also be implications for flora and fauna in the area. These implications would be more detrimental around Site 1 than the other two sites given the rather small difference for most metals between Sites 2 and 3 and the control site. These small differences, however, may also indicate that even at the distance chosen for the control site to any of the Au mines, there is a risk related to metal contaminated soils and that much larger distances would need to be considered for finding a true reference point for uncontaminated soils in future studies.

### Human exposure to heavy metals and metalloids around tailings dumps

Based on the daily intake values used in this study, daily intake of the heavy metals and metalloids at all three sites was higher among children than among adults for all three pathways of exposure (Table 4). Average daily intake was highest through ingestion, followed by dermal and finally by inhalation pathway. At Site 1, daily intake was highest for Zn and lowest for Cu. At Sites 2 and 3, Cr daily intake values were highest. Average daily intake of As was lowest at Site 3, whereas at Site 2, Cd had the lowest average daily intake value. Details of the average daily intake values for each exposure pathway for heavy metals and metalloids at the different sites are presented in Table 4. Non-carcinogenic hazard quotients (*HQ*) of heavy metals and metalloids at the sites were higher among children than among adults (Table 4). At all three sites, *HQ* values for exposure through inhalation was <1 for both adults and children. At Site 2 and Site 3, *HQ* values for Cr were >1 for children for ingestion exposure, and for both adults and children through dermal exposure (Table 4).

**Table 4:** Average daily intake of heavy metals and metalloids by adults and children and the respective hazard quotients.

Heavy metal	Site	Daily intake (mg/kg bw/d)						Hazard quotient (HQ)					
		Ingestion		Inhalation		Dermal		Ingestion		Inhalation		Dermal	
		adult	child	adult	child	adult	child	adult	child	adult	child	adult	child
As	1	1.7E-03	1.6E-02	2.5E-07	4.5E-07	5.9E-04	2.0E-03	5.8E+00	5.4E+01	1.7E-02	3.0E-02	4.8E+00	1.6E+01
	2	2.1E-05	2.0E-04	3.1E-09	5.5E-09	7.3E-06	2.4E-05	7.1E-02	6.6E-01	2.1E-04	3.7E-04	5.9E-02	2.0E-01
	3	5.7E-06	5.3E-05	8.4E-10	1.5E-09	2.0E-06	6.4E-06	1.9E-02	1.8E-01	5.6E-05	9.9E-05	1.6E-02	5.2E-02
Cd	1	1.5E-05	1.4E-04	2.2E-09	3.9E-09	5.1E-06	1.7E-05	1.5E-02	1.4E-01	2.2E-04	3.9E-04	5.1E-01	1.7E+00
	2	7.1E-06	6.6E-05	1.0E-09	1.8E-09	2.4E-06	8.0E-06	7.1E-03	6.6E-02	1.0E-04	1.8E-04	2.4E-01	8.0E-01
	3	1.7E-05	1.6E-04	2.5E-09	4.4E-09	5.8E-06	1.9E-05	1.7E-02	1.6E-01	2.5E-04	4.4E-04	5.8E-01	1.9E+00
Co	1	2.0E-03	1.9E-02	3.0E-07	5.3E-07	6.9E-04	2.3E-03	1.0E-01	9.4E-01	5.2E-02	9.2E-02	4.3E-02	1.4E-01
	2	8.2E-06	7.6E-05	1.2E-09	2.1E-09	2.8E-06	9.2E-06	4.1E-04	3.8E-03	2.1E-04	3.7E-04	1.8E-04	5.8E-04
	3	8.8E-06	8.2E-05	1.3E-09	2.3E-09	3.0E-06	9.9E-06	4.4E-04	4.1E-03	2.3E-04	4.0E-04	1.9E-04	6.2E-04
Cr	1	2.6E-04	2.4E-03	3.8E-08	6.8E-08	8.9E-05	2.9E-04	8.6E-02	8.1E-01	1.3E-03	2.4E-03	1.5E+00	4.9E+00
	2	7.8E-04	7.3E-03	1.2E-07	2.0E-07	2.7E-04	8.9E-04	2.6E-01	2.4E+00	4.0E-03	7.1E-03	4.5E+00	1.5E+01
	3	3.7E-04	3.5E-03	5.5E-08	9.8E-08	1.3E-04	4.2E-04	1.3E-01	1.2E+00	1.9E-03	3.4E-03	2.1E+00	7.1E+00
Cu	1	1.7E-03	1.6E-02	2.5E-07	4.5E-07	5.9E-04	2.0E-03	4.3E-02	4.0E-01	6.3E-06	1.1E-05	5.0E-02	1.6E-01
	2	2.5E-05	2.4E-04	3.7E-09	6.6E-09	8.6E-06	2.8E-05	6.3E-04	5.9E-03	9.2E-08	1.6E-07	7.2E-04	2.4E-03
	3	5.9E-05	5.5E-04	8.7E-09	1.5E-08	2.0E-05	6.7E-05	1.5E-03	1.4E-02	2.2E-07	3.8E-07	1.7E-03	5.6E-03
Mn	1	4.3E-03	4.0E-02	6.3E-07	1.1E-06	1.5E-03	4.9E-03	9.3E-02	8.7E-01	4.4E-02	7.8E-02	5.1E-01	1.7E+00
	2	2.4E-04	2.2E-03	3.5E-08	6.2E-08	8.2E-05	2.7E-04	5.2E-03	4.8E-02	2.5E-03	4.3E-03	4.4E-02	1.5E-01
	3	3.1E-04	2.9E-03	4.6E-08	8.2E-08	1.1E-04	3.6E-04	6.8E-03	6.4E-02	3.2E-03	5.7E-03	5.9E-02	1.9E-01
Ni	1	4.8E-03	4.5E-02	7.0E-07	1.2E-06	1.6E-03	5.4E-03	2.4E-01	2.2E+00	2.7E-05	4.8E-05	3.0E-01	1.0E+00
	2	5.0E-05	4.7E-04	7.4E-09	1.3E-08	1.7E-05	5.7E-05	2.5E-03	2.3E-02	2.8E-07	5.0E-07	3.2E-03	1.1E-02
	3	9.1E-05	8.5E-04	1.3E-08	2.4E-08	3.1E-05	1.0E-04	4.6E-03	4.2E-02	5.1E-07	9.1E-07	5.8E-03	1.9E-02
Pb	1	7.1E-05	6.7E-04	1.1E-08	1.9E-08	2.5E-05	8.1E-05	2.0E-02	1.9E-01	3.0E-06	5.3E-06	4.7E-02	1.5E-01
	2	5.2E-05	4.8E-04	7.6E-09	1.4E-08	1.8E-05	5.9E-05	1.5E-02	1.4E-01	2.2E-06	3.8E-06	3.4E-02	1.1E-01
	3	4.8E-05	4.5E-04	7.0E-09	1.3E-08	1.6E-05	5.4E-05	1.4E-02	1.3E-01	2.0E-06	3.5E-06	3.1E-02	1.0E-01
Zn	1	6.7E-03	6.2E-02	9.8E-07	1.7E-06	2.3E-03	7.6E-03	2.2E-02	2.1E-01	3.3E-06	5.8E-06	8.0E-01	2.6E+00
	2	1.1E-04	1.0E-03	1.6E-08	2.9E-08	3.8E-05	1.2E-04	3.6E-04	3.4E-03	5.4E-08	9.5E-08	6.3E-04	2.1E-03
	3	2.3E-04	2.1E-03	3.3E-08	5.9E-08	7.8E-05	2.6E-04	7.6E-04	7.1E-03	1.1E-07	2.0E-07	1.3E-03	4.3E-03

**Fig 8:**
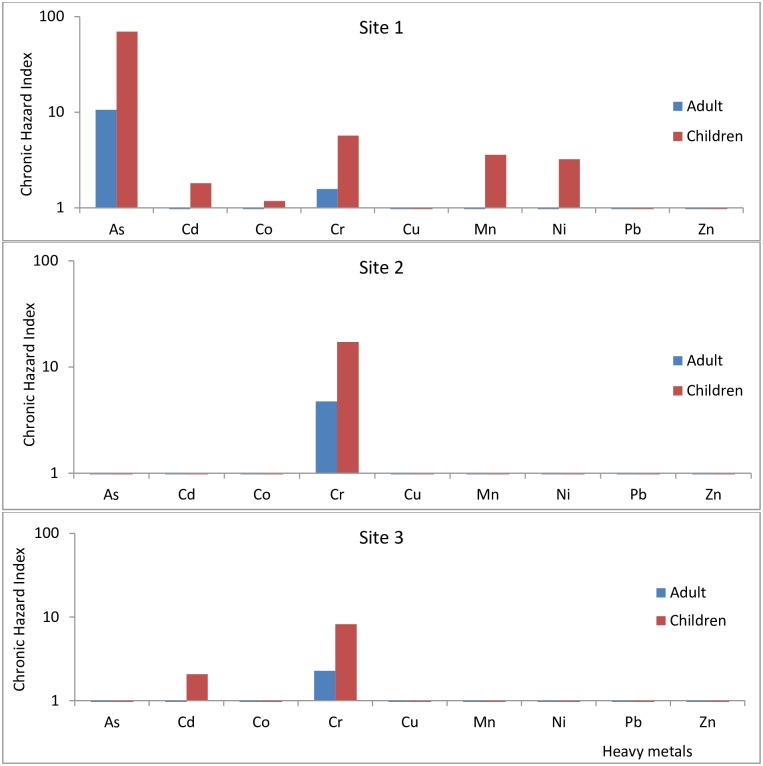
Chronic hazard index (*CHI*) for each metal and metalloid at the three study sites.

Lower exposure at Site 2 and Site 3 compared to Site 1 can be attributed to the lower levels of heavy metals and metalloid at these sites. At Site 3, children are significantly exposed to both Cd and Cr whereas adults are exposed to Cr only (Fig 8). These results are in line with screening level assessment which indicate high non-carcinogenic risk per emission unit for As, followed by Cd, Zn, Pb, Ni, Cr and Cr in decreasing order based on human toxicity characterization factors obtained with USEtox (Fig 7).

Significant exposure risk exists at Site 1 where values for *HQ* and *CHI* for As, Cd, Co, Cr, Ni, and Mn in children and As and Cr for adults were > 1 (Fig 8). Non-carcinogenic risk associated with exposure of humans to heavy metals and metalloid in soils was therefore greatest at Site 1 followed by Site 3 and finally Site 2. Numerous studies explaining the health effects associated with exposure of humans to heavy metals exist [65–67]. Results obtained in this study indicate that there is significant heavy metal and metalloid exposure risk at Site 1 compared to the other two sites.

### Carcinogenic risks associated with heavy metals and metalloids exposure at the three sites

Screening-level human toxicity results using USEtox indicate that exposure to some heavy metals and metalloids like Pb, Ni, Cd, and As could result in potential cancer risk (Fig 7). A detailed carcinogenic risk assessment of the different samples indicates that carcinogenic risk for the different metals varied with sites. Exposure of individuals to soils from Site 1 presented a significantly higher cancer risk for As and Ni compared to the other sites. Arsenic cancer risk (*CR*) values were 2.6x10^-3^ (*CR*_*Ing*_ adult), 2.4x10^-2^ (*CR*_*Ing*_ children), 2.2x10^-3^ (*CR*_*dermal*_ adult), 7.1x10^-3^ (*CR*_*dermal*_ children), 4.8x10^-3^ (Total Cancer Risk adult) and 3.1x10^-2^ (*TCR* children). Cancer risk values for Ni at Site 1 was 4.0x10^-3^ (*CR*_*Ing*_ adult), and 3.7x10^-2^ (*CR*_*Ing*_ children). Cancer risk values for As and Cd at Site 1 were all higher than 1x10^-4^ which is the unacceptable risk threshold. At Site 2, carcinogenic risk value for As through ingestion by children was 2.9x10^-4^ and for Ni, 3.9x10^-4^ with a total carcinogenic risk of 3.9x10^-4^ for As and Ni in children. Carcinogenic risk at Site 3 was lowest with values for cancer related risk associated with Ni in children through ingestion being 7.1x10^-4^. There was no significant carcinogenic risk for adults at Site 2 as all *TCR* values were < 1x10^-4^. At Site 3 there is a possibility of children ingesting soils and tailing particles developing cancer related health risk associated with Ni (Ni *CR*_*Ing*_ = 7.1x10^-4^). All other values for this site were less than the unacceptable value of 1x10^-4^.

### Ecological and human health Implications of exposure to tailings contaminated soil

The effects of prolonged exposure of various fauna and flora species to heavy metals have been reported by Fayiga et al. [68], Franco-Hernández et al [69], Sethy and Ghosh [70], Chibuike and Obiora [14], Emamverdian et al. [71], Sobelev and Begonia [15], Migliorini et al. [16], Gall et al. [17] and Eisler, [18]. Results from our study have highlighted the potential ecological risk which exists around some mine tailings in the study area. Ecosystems around Au mine tailings dump are at a huge risk from heavy metals and metalloids contained in the tailings. This risk varies with site and heavy metal or metalloid in question. In this study, As, Co and Ni presented the greatest ecological risk. The effect of As on birds and mammals has been presented by Eisler [18]. According to Finnegan and Chen [72], plant growth is stimulated by low concentrations of As, since few plants are tolerant to high concentrations of the metal. Nickel at low concentrations also has a vital role in various plant morphological and physiological functions [73], but high Ni concentrations affects lateral root development, photosynthesis, mineral nutrition and enzymatic activity and would therefore affect vegetation growth. Cobalt and Zn would also affect seed germination. High concentrations of these heavy metals would therefore affect the development of any vegetation cover in the area, preventing OM accumulations in the soil, which would otherwise serve as a source for nutrients for soil biota. The ecosystem functioning and diversity will therefore be greatly affected especially at site 1. This is not only for terrestrial ecosystem as heavy metals in tailings could also find their way into surface water systems as indicated in Fig 1. The potential toxicity of these heavy metals and metalloids to freshwater ecosystems will also vary with site. Using USEtox, the freshwater ecotoxicity characterization factors were highest for Pb and lowest for Cu (Fig 7). No freshwater body is close to Sites 2 and 3, but there is a dam in the precincts of Site 1. Considering that the concentrations of all heavy metals were highest in soils at Site 1, dispersal of mine contaminated soil into the dam is likely to increase the freshwater ecotoxicity factors.

High exposure of humans to As is associated with skin damage, increased risk of cancer, and problems with circulatory system [74]. Cadmium and Pb are carcinogenic and prolonged exposure to low concentrations could lead to kidney disease, lung damage, and fragile bones for Cd, and nervous disorder in the case of Pb. Cobalt has been implicated in cardiomyopathy and lung irritation. According to ATSDR [19], Cr may cause gastrointestinal, nasal and lung irritation, ulceration of stomach and small intestines, dermatitis, and decreased sperm counts. Dermatitis, lung inflammation and cancer are health conditions associated with excessive Ni intake. The most commonly reported adverse health effects of Cu and Zn are gastrointestinal distress including nausea, vomiting, and/or abdominal pain but irritation of the respiratory system is also common [44]. These complications are likely to be experienced by residents close to site 1 because of the higher concentrations of heavy metals and metalloids at this site compared to the others. In addition, screening-level human toxicity and freshwater ecosystem toxicity assessment using USEtox (Fig 6) indicates ingestion of above-ground crops contributes significantly to exposure to Cd, Cu, Mn, Pb and Zn (Fig 6). Considering that vegetables destined for human consumption are grown on these soils by some residents, total daily intake of the metals could actually be higher than what is presented in our study. Research in South Africa has shown that HIV is more prevalent among informal settlements [24] and these communities are the ones living close to tailings dumps from mining activities. The consequences of heavy metal exposure in these communities would be more grievous because of an already compromised immune system.

The extent to which individuals exposed to these metals and metalloids are affected is also influenced by several factors among which include the species of the heavy metal or metalloid present, the mineralogy of the soil as well as the environment in the gastrointestinal tract (in the case of oral exposure) [75]. Studies by Tahiri et al, [76] show that under fed conditions, dissolved organic matter which may readily form a complex with some metals like Pb is present in the GIT, and could reduce metal bioaccessibility, their uptake and consequently the negative effect on the exposed individual. In addition, precipitation or chelation under the prevailing alkaline conditions in the stomach has also been reported to reduce bioaccessibility of metals in the stomach [77]. The kinds of food and microorganisms present in the GIT may reduce absorption through chelation processes [77],[78]. GIT microflora like Lactobacillus and Bifidobacterium are known to bind Cd, Pb and other metals in solution [78]. These factors are however not usually taken into consideration in the determination of metal and metalloid exposure risk.

## Conclusion

Abandoned gold mine tailings present serious ecological and human health risk associated with exposure to heavy metals and metalloids emitted into the environment. The findings of this study indicate that high concentrations of As, Cd, Co, and Ni in soil may present unacceptable risks to human populations as well as to ecosystems in the vicinity of mining areas. The extent to which ecosystems may be affected varies from one Au mine to the other. Humans in the vicinity of mine tailings are at risk of developing cancer and non-cancer health complications associated with exposure to heavy metals via several ingestion- and dermal-related pathways and via inhalation. The exposure-related risks are higher among children than adults, mainly via ingestion and dermal exposure. Communities in the vicinity of tailings should take precaution to reduce the frequency of exposure to these soils in order to reduce the probability of developing a health complication. Efforts to prevent consumption of vegetables grown on these soils should be intensified to reduce related risks on humans, and improved methods for combining measured metal concentrations in contaminated soils with toxicity characterization modeling are required to provide further insights and to identify and evaluate emission and exposure reduction measures.

## Supporting information

S1 Table Concentrations of heavy metals and metalloids in replicate samples from each site. (DOCX)
