# Supplementary material for: Ecological and human health risks associated with abandoned gold mine tailings contaminated soil
Source: PLoS One. 2017 Feb 21;12(2):e0172517. doi: 10.1371/journal.pone.0172517 (PMC5319768; doi:10.1371/journal.pone.0172517)
Supplement: S1 Table — (DOCX) [file pone.0172517.s001.docx]

S1 Table. Concentrations of Heavy metals and metalloids in replicate samples from each site

| Site | replicates | Concentration in mg/kg | | | | | | | | |
| --- | --- | --- | --- | --- | --- | --- | --- | --- | --- | --- |
|  |  | As | Cd | Co | Cr | Cu | Mn | Ni | Pb | Zn |
| Control | sample 1 | 4.00 | 2.60 | 3.60 | 106.60 | 28.98 | 171.40 | 20.40 | 9.30 | 81.90 |
|  | sample 2 | 3.20 | 2.90 | 4.90 | 134.60 | 33.70 | 166.70 | 21.20 | 18.00 | 81.05 |
|  | sample 3 | 3.05 | 3.02 | 5.26 | 124.14 | 44.43 | 173.19 | 21.72 | 17.60 | 94.57 |
|  | sample 4 | 2.95 | 3.10 | 4.25 | 107.29 | 20.84 | 162.09 | 19.36 | 14.21 | 82.61 |
|  | sample 5 | 3.62 | 2.50 | 3.79 | 118.08 | 19.50 | 161.48 | 24.44 | 15.52 | 82.45 |
|  | sample 6 | 3.04 | 2.85 | 5.35 | 141.36 | 40.42 | 157.33 | 26.87 | 20.39 | 91.67 |
|  | sample 7 | 4.00 | 3.22 | 5.12 | 128.02 | 34.54 | 165.99 | 23.47 | 18.01 | 91.03 |
|  | sample 8 | 2.79 | 3.17 | 5.65 | 139.48 | 42.49 | 164.56 | 32.66 | 21.22 | 93.20 |
|  | sample 9 | 3.35 | 3.17 | 4.86 | 127.28 | 29.49 | 166.00 | 25.24 | 17.89 | 91.17 |
|  | sample 10 | 3.61 | 2.48 | 5.39 | 132.67 | 37.43 | 164.76 | 31.80 | 21.78 | 105.86 |
|  | sample 11 | 3.17 | 3.09 | 5.26 | 127.72 | 34.10 | 165.89 | 24.74 | 17.94 | 86.53 |
|  | sample 12 | 3.35 | 2.49 | 5.61 | 138.74 | 41.67 | 163.46 | 23.28 | 21.37 | 101.21 |
| Site 1 | sample 1 | 1038.89 | 10.19 | 1399.07 | 167.32 | 1128.70 | 2601.85 | 3974.07 | 72.46 | 5339.81 |
|  | sample 2 | 917.75 | 8.20 | 1267.17 | 142.25 | 1151.67 | 2435.83 | 1733.33 | 42.40 | 5095.00 |
|  | sample 3 | 1014.45 | 7.30 | 1068.10 | 159.50 | 1194.50 | 2192.61 | 1336.97 | 39.25 | 3699.16 |
|  | sample 4 | 1313.21 | 11.41 | 1572.64 | 191.89 | 1310.38 | 2752.64 | 4040.57 | 46.51 | 2203.77 |
|  | sample 5 | 1401.05 | 9.97 | 1679.94 | 131.34 | 1334.39 | 3012.00 | 1076.00 | 46.79 | 3871.53 |
|  | sample 6 | 1287.14 | 10.24 | 1489.79 | 105.39 | 1235.41 | 2984.20 | 3527.21 | 48.34 | 2843.56 |
|  | sample 7 | 1010.42 | 10.52 | 1266.84 | 201.69 | 1024.35 | 2970.10 | 3634.81 | 48.28 | 4946.96 |
|  | sample 8 | 1493.12 | 10.79 | 1394.87 | 167.99 | 1204.39 | 3083.22 | 2164.92 | 46.62 | 4842.64 |
|  | sample 9 | 1254.63 | 9.07 | 1574.41 | 208.75 | 1294.03 | 3203.23 | 1770.66 | 46.51 | 4321.39 |
|  | sample 10 | 1034.65 | 11.35 | 1165.36 | 242.05 | 1152.47 | 2601.60 | 2736.51 | 46.59 | 4801.91 |
|  | sample 11 | 1138.72 | 7.62 | 1211.16 | 145.47 | 1062.64 | 3443.24 | 3731.10 | 49.44 | 3465.33 |
|  | sample 12 | 974.15 | 11.90 | 1364.02 | 106.00 | 1350.35 | 3563.25 | 2261.27 | 46.01 | 4895.59 |
| Site 2 | sample 1 | 16.29 | 2.73 | 3.42 | 200.90 | 13.30 | 87.21 | 7.90 | 10.78 | 69.74 |
|  | sample 2 | 17.30 | 2.54 | 3.63 | 106.56 | 18.98 | 61.40 | 18.30 | 5.30 | 81.90 |
|  | sample 3 | 4.05 | 1.08 | 3.51 | 140.20 | 19.88 | 169.90 | 16.42 | 0.39 | 79.80 |
|  | sample 4 | 3.09 | 1.80 | 0.88 | 14.02 | 5.85 | 11.35 | 0.85 | 28.16 | 81.87 |
|  | sample 5 | 5.62 | 1.67 | 0.43 | 9.65 | 5.60 | 127.49 | 8.38 | 10.01 | 82.20 |
|  | sample 6 | 14.53 | 1.11 | 1.93 | 267.45 | 13.09 | 100.62 | 7.93 | 11.95 | 52.72 |
|  | sample 7 | 8.55 | 4.82 | 4.31 | 249.93 | 7.65 | 103.67 | 19.06 | 9.75 | 71.06 |
|  | sample 8 | 12.70 | 4.52 | 2.25 | 236.96 | 18.55 | 106.73 | 17.22 | 10.05 | 80.47 |
|  | sample 9 | 6.93 | 8.42 | 3.48 | 198.14 | 14.61 | 109.78 | 11.44 | 14.62 | 62.42 |
|  | sample 10 | 15.61 | 3.13 | 5.89 | 153.77 | 4.01 | 112.83 | 19.58 | 13.29 | 70.94 |
|  | sample 11 | 15.92 | 3.22 | 5.72 | 171.89 | 15.58 | 115.88 | 26.30 | 9.44 | 75.06 |
|  | sample 12 | 6.13 | 5.65 | 3.88 | 184.22 | 17.39 | 118.93 | 23.66 | 13.34 | 80.25 |
| Site 3 | sample 1 | 4.00 | 0.20 | 5.00 | 210.67 | 36.70 | 99.04 | 1.84 | 28.80 | 47.10 |
|  | sample 2 | 3.10 | 0.03 | 4.00 | 214.57 | 33.18 | 199.69 | 2.50 | 23.60 | 46.87 |
|  | sample 3 | 2.10 | 0.20 | 7.70 | 287.70 | 53.40 | 196.90 | 33.10 | 44.30 | 194.40 |
|  | sample 4 | 0.40 | 0.20 | 5.00 | 344.60 | 43.70 | 192.00 | 30.18 | 21.10 | 175.00 |
|  | sample 5 | 3.95 | 0.20 | 3.61 | 240.35 | 26.33 | 289.00 | 30.70 | 25.99 | 49.00 |
|  | sample 6 | 3.20 | 0.20 | 6.92 | 235.03 | 44.48 | 176.00 | 13.03 | 24.89 | 167.00 |
|  | sample 7 | 2.70 | 1.00 | 6.90 | 276.35 | 26.27 | 186.00 | 21.12 | 29.65 | 185.00 |
|  | sample 8 | 1.80 | 3.00 | 4.62 | 178.29 | 27.24 | 65.00 | 27.75 | 23.57 | 190.00 |
|  | sample 9 | 1.30 | 2.00 | 8.71 | 248.93 | 37.12 | 191.00 | 24.13 | 35.18 | 65.00 |
|  | sample 10 | 2.80 | 6.00 | 5.10 | 262.94 | 35.81 | 201.00 | 33.46 | 38.79 | 69.00 |
|  | sample 11 | 2.90 | 0.10 | 3.52 | 213.38 | 43.39 | 211.00 | 29.92 | 28.41 | 89.00 |
|  | sample 12 | 3.00 | 0.20 | 3.81 | 270.32 | 13.37 | 189.00 | 27.72 | 42.60 | 106.00 |
